# High Symptom Burden in Patients With Advanced Chronic or Prolonged Infectious Diseases: Not Only Pain

**DOI:** 10.7759/cureus.71751

**Published:** 2024-10-18

**Authors:** Elena Angeli, Agostino Zambelli, Oscar Corli, Giovanna Bestetti, Simona Landonio, Stefania Merli, Stefania Cheli, Giuliano Rizzardini

**Affiliations:** 1 Infectious Diseases Department, Fatebenefratelli-Sacco University Hospital, Milan, ITA; 2 Pain Therapy and Palliative Care Unit, Institute for Pharmacological Research Mario Negri, Istituto di Ricovero e Cura a Carattere Scientifico (IRCCS), Milan, ITA; 3 Pharmacovigilance and Clinical Research, Biomedical and Clinical Sciences Department, Fatebenefratelli-Sacco University Hospital, Milan, ITA

**Keywords:** chronicity, edmonton symptom assessment system, infectious diseases, palliative medicine, symptom burden

## Abstract

Introduction: The growing evidence of increased life expectancy in the future reveals the high relevance of frailty in patients with chronic-degenerative diseases; identification and management of symptoms may improve significantly their quality of life. The objective of our study was to assess the symptom burden in patients with advanced chronic or prolonged infectious diseases.

Materials and methods: A cross-sectional study was performed enrolling 88 patients, referred to palliative care consultation for chronic pain, and evaluated using the Edmonton Symptom Assessment System to define Total Symptom Distress Score (TSDS) and high symptom burden (HSB) when more than six symptoms along with Numerical Rating Scale ≥4 were present.

Results: All participants reported moderate to severe pain; in addition, 86 (97.7%) experienced a lack of well-being, 81 (92%) tiredness, 67 (76.1%) lack of appetite, 66 (75%) drowsiness, 66 (75%) depression, 56 (63.6%) anxiety, 49 (55.6%) nausea, and 39 (44.3%) shortness of breath. Forty-four patients (50%) had high TSDS, greater than 40.5, and presented lower Karnofsky Performance Scale (KPS) (median 40 vs. 70, p=0.0005), higher comorbidities (median 7 vs. 4, p=0.00001), and higher drug burden (median 9 vs. 6, p=0.0003) than those with low TSDS. Furthermore, considering symptom intensity, 40 patients (45.4%) had HSB and presented lower KPS (median 50 vs. 70, p=0.0005), higher comorbidities (median 7 vs. 4, p=0.00001), and higher drug burden (mean 9 vs. 6, p=0.01) compared to patients without HSB.

Conclusion: Our population had an HSB, in addition to pain, revealing high frailty. A correct assessment of symptoms is, therefore, required to manage patients with chronic infectious diseases. In this setting, attention should be given to identifying patients at high risk of HSB through a correct diagnosis and effective management, which should be based on a multi-professional approach.

## Introduction

The longer life expectancy for the population in the future reveals the growing impact of frailty in patients with chronic degenerative diseases. In this setting, understanding symptoms and giving the right answers to patients’ needs are crucial to improving their quality of life. This topic has been fully investigated in patients with cancer [1,2], but in the non-oncologic population, it is limited to a few conditions, mainly in patients with end-stage cardiac, pulmonary, and liver diseases [3-6] and dementia [7]. In patients with infectious diseases, this topic has been explored quite exclusively in people living with HIV (PLWH) [8] and, more recently, with long COVID [9]. In addition, in these conditions, attention is mostly addressed to the diagnosis and management of chronic pain, whereas concomitant symptoms are often underestimated and not adequately considered, with a negative impact on quality of life and on the efficacy of pain treatment itself. Pain remains the most frequently reported symptom by patients for its impact on physical, social, and psychological domains, with the risk of substantial disability and increased limitation in daily activities. In the setting of chronic infectious diseases, it has been reported that in PLWH, chronic pain is present in 54-83% of cases [10]. It is well known, for example, that the concomitant presence of depressive symptoms has been documented to contribute to increased pain [11,12]. In addition, effective treatment of pain is sometimes related to the improvement of psychological symptoms, underscoring that the emotional-psychological component of pain is extremely important [13,14]. Therefore, only the correct diagnosis of all symptoms using specific instruments can lead to a complete understanding of patients’ needs in order to direct them to the appropriate treatment.

Several tools have been used to evaluate the complexity of symptoms in patients affected by cancer or chronic diseases; among them, one of the first and most widely used is the Edmonton Symptoms Assessment System (ESAS) [15], which assesses the presence and intensity of pain and other concomitant symptoms, such as asthenia, nausea, depression, anxiety, drowsiness, lack of appetite, dyspnea, and lack of wellbeing, ranging from absent (Numerical Rating Scale (NRS) 0), mild (NRS 1-3), moderate (NRS 4-6), and severe (NRS 7-10). ESAS has been recently applied to PLWH, revealing that pain, asthenia, and depression were frequent and that symptom intensity was related to the presence of comorbidities [16].

The present study examined the complexity and intensity of symptoms in patients affected by advanced chronic or prolonged infectious diseases to identify those at risk of presenting a high symptom burden (HSB). The objectives for this study were (1) to identify and score physical and emotional symptoms using the ESAS and (2) to analyze clinical differences among patients with or without an HSB.

## Materials and methods

Study design and participants

This is a cross-sectional study that evaluated patients affected by advanced chronic or prolonged infectious diseases and referred to a palliative care consultation for the presence of chronic pain. We included patients affected by chronic infection when infection disease was present for more than six months and patients with prolonged infections when symptoms were persistent after acute infection for more than one month. Between January 2022 and November 2023, participants were consequently recruited from the Department of Infectious Diseases of a primary care university hospital in Milan, Italy.

Inclusion and exclusion criteria

Individuals were considered eligible if they (1) were affected by an advanced chronic or prolonged infectious disease, (2) were aged 18 or older, (3) reported chronic pain, (4) signed the informed consent, and (5) were able to understand and fill out the ESAS questionnaire. Individuals were considered ineligible if they (1) had a poor life expectancy (i.e., less than one year), (2) had concomitant active and painful comorbidities, and (3) refused to sign informed consensus.

Ethical considerations

This study was conducted in accordance with the Declaration of Helsinki and the Guidelines of Good Clinical Practice issued by the International Council for Harmonization of Technical Requirements for Pharmaceuticals for Human Use. It was approved by Comitato Etico Milano Area 1 (approval number: 2023/ST/004).

Data collection

Eligible individuals were recruited during the first clinical evaluation at the palliative care clinic, as requested due to the presence of pain by the infectious diseases care provider. For each patient, clinical-demographic data about age, gender, BMI, Karnofsky Performance Scale (KPS), comorbidities, active therapeutic burden, and infectious disease diagnosis were collected.

After providing consent, patients were adequately informed about how to complete the ESAS questionnaires. Each participant was requested to fill out the questionnaire, rating the intensity score for each symptom, ranging from 0 (absent) to 10 (worst). To assess average symptom severity, participants were asked to indicate the numeric value, from 0 to 10, that best described their symptom on average during the previous 24 hours.

Total Symptom Distress Score (TSDS) was obtained by summing each symptom score. In detail, physical TSDS is derived by the sum of scores of pain, tiredness, nausea, drowsiness, lack of appetite, shortness of breath, and lack of well-being, whereas emotional TSDS includes scores of depression and anxiety together. Another optional symptom, such as constipation, diarrhea, sleeplessness, etc., was added if the patient reported it. We defined patients with HSB as those who reported more than six symptoms with an intensity score equal to or greater than 4.

Sample size

A previously published study found a significant correlation between the effect of real-time electronic monitoring of patient-reported symptoms and the improvement in the ESAS score [17]. Specifically, they found a significant difference of 5.70 (95% CI 1.96, 9.43) in ESAS score in favor of the intervention arm. Assuming that similar results can be obtained in our studies, a minimum of 58 patients will be considered sufficient to detect statistically significant differences with a power of 80% and a confidence interval of 95%.

Statistical analysis

The analyses are mostly descriptive. Quantitative variables were expressed as mean and standard deviation (SD) or median and interquartile range (IQR), according to the data distribution verified using the Kolmogorov-Smirnov test. Qualitative variables were described as frequencies and percentages for each category. Comparisons between groups were performed by the Student's t-test or Mann-Whitney U test for quantitative variables and the χ2 test for qualitative variables. Statistical significance was defined as a two-sided p-value <0.05.

A qualitative and quantitative analysis of symptoms derived from the ESAS questionnaire was performed. Mild (scored 1-3), moderate (scored 4-6), and severe (scored 7-10) ESAS scores were assigned. For analysis, TSDS ratings were divided into the binary categories of "high" (HTSDS) and "low" (LTSDS) based on calculating each patient's median TSDS score. Accordingly, each patient's symptom burden score was classified into two categories: low symptom burden and HSB, respectively, when less than 6 and equal to or greater than 6 symptoms with an intensity score NRS ≥4 were simultaneously present. Univariate analyses were then conducted to determine the association between symptom scores (TSDS, HSB) and patient-related variables (age, gender, BMI, KPS, comorbidities, and co-medications).

## Results

Participant clinical demographics

During the study, 108 subjects received specialist consultation in palliative care and were screened for enrollment. Among them, 88 patients satisfied the inclusion criteria, whereas 20 were excluded. Participants were predominantly male (53.4%), with a mean age of 69±14 years, a mean BMI of 25.2±5.1, and a median KPS of 60 (IQR 40-80).

Almost all participants had one or more comorbidities, with a median of 5 (IQR 3-7) and a median therapeutic burden of 8 (IQR 5-10) concomitant drugs (Table 1). Considering the infectious disease diagnosis, 39 patients were affected by osteomyelitis or prosthetic joint infection, 17 had HIV infection, 15 had chronic infected wounds, 10 had severe and persistent herpes-zoster infection, three had HBV or HCV-related liver cirrhosis, three had recurrent sepsis, and one suffered from long-term COVID.

**Table 1:** Main features of the 88 enrolled patients N: total number of patients, n: number of patients, SD: standard deviation, BMI: body mass index, KPS: Karnofsky Performance Scale, IQR: interquartile range

Participants N°	N=88
Female (%)	41 (46.6)
Male (%)	47 (53.4)
Age (mean±SD), years	69±14
BMI (mean±SD)	25.2±5.1
KPS (median; IQR)	60; 40-80
Comorbidities (median; IQR)	5; 3-7
n° (%) <3	23 (26.1)
n° (%) 4-6	35 (39.8)
n° (%) >7	30 (34.1)
Drug burden (median; IQR)	8; 5-10
n° (%) <3	14 (15.9)
n° (%) 4-6	21 (23.9)
n° (%) >7	53 (60.2)

ESAS analysis

All participants reported pain, with 11 (12.5%) with NRS 4-6 and 77 (87.5%) with NRS 7-10. Other than pain, most of the patients complained of one or more symptoms simultaneously. In particular, considering physical ESAS, 86 patients (97.7%) presented a lack of well-being, 81 (92%) tiredness, 67 (76.1%) lack of appetite, 66 (75%) drowsiness, 49 (55.6%) nausea, and 39 (44.3%) shortness of breath (Table 2). For emotional ESAS, 66 (75%) patients reported depression and 56 (63.6%) anxiety (Table 3). Eighteen patients (20.4%) indicated other optional symptoms, such as constipation (14), diarrhea (2), and sleeplessness (2).

**Table 2:** Frequency and intensity of ESAS physical symptoms of the 88 participants N: total number of patients, n: number of patients, ESAS: Edmonton Symptoms Assessment System, NRS: Numerical Rating Scale

ESAS physical symptoms	n/N (%)
Pain	88/88 (100)
NRS 0	0 (0)
NRS 1-3	0 (0)
NRS 4-6	11 (12.5)
NRS 7-10	77 (87.5)
Lack of well-being	86/88 (97.7)
NRS 0	2 (2.3)
NRS 1-3	3 (3.4)
NRS 4-6	20 (22.7)
NRS 7-10	63 (71.6)
Tiredness	81/88 (92)
NRS 0	7 (8)
NRS 1-3	11 (12.5)
NRS 4-6	19 (21.6)
NRS 7-10	51 (57.9)
Lack of appetite	67/88 (76.1)
NRS 0	21 (23.9)
NRS 1-3	15 (17)
NRS 4-6	30 (34.1)
NRS 7-10	22 (25)
Drowsiness	66/88 (75)
NRS 0	22 (25)
NRS 1-3	13 (14.8)
NRS 4-6	22 (25)
NRS 7-10	31 (35.2)
Nausea	49/88 (55.6)
NRS 0	39 (44.3)
NRS 1-3	25 (28.4)
NRS 4-6	17 (19.3)
NRS 7-10	7 (8)
Shortness of breath	39/88 (44.3)
NRS 0	50 (56.8)
NRS 1-3	20 (22.7)
NRS 4-6	16 (18.2)
NRS 7-10	2 (2.3)

**Table 3:** Frequency and intensity of ESAS emotional symptoms of the 88 participants N: total number of patients, n: number of patients, ESAS: Edmonton Symptoms Assessment System, NRS: Numerical Rating Scale

ESAS emotional symptoms	n/N (%)
Depression	66/88 (75%)
NRS 0	22 (25)
NRS 1-3	13 (14.8)
NRS 4-6	22 (25)
NRS 7-10	31 (35.2)
Anxiety	56/88 (63.6%)
NRS 0	32 (36.3)
NRS 1-3	13 (14.8)
NRS 4-6	21 (23.9)
NRS 7-10	22 (25)

TSDS and HSB quantification

TSDS was calculated for each patient; the median TSDS was 40.5 (IQR 29-50). We stratified patients according to HTSDS if they had equal or more than 40.5 and LTSDS for those with less than 40.5. Forty-four (50%) patients had HTSDS and 44 (50%) LTSDS. After univariate analysis, patients with HTSDS had significantly lower KPS (median 40, IQR 40-60 vs. 70, IQR 50-90; p=0.0005), a higher number of comorbidities (median 7, IQR 4-9 vs. 4, IQR 3-5.3; p=0.00001), and a higher drug burden (median 9, IQR 8-11 vs. 6, IQR 3.8-8; p=0.0003). According to their symptom burden score, we compared patients with HSB to those with LSB; we found that patients with HSB were significantly different for lower KPS (median 50, IQR 40-60 vs. 70, IQR 50-90; p=0.0005), higher number of comorbidities (median 7, IQR 4-9 vs. 4, IQR 3-5; p=0.00001) and higher drug burden (median 9, IQR 7.3-11 vs. 6, IQR 4-8; p=0.01).

## Discussion

In this study, we investigated the prevalence and intensity of symptoms and the correlation with clinical parameters in patients with advanced chronic or persistent infectious diseases. The majority of our patients complained of multiple symptoms, in addition to pain, which typically represents the pivotal sign that leads to palliative care consultation. This topic is currently underexplored and limited to specific settings, such as PLWH and those with end-stage liver diseases, which included mostly patients with hepatocellular carcinoma and so affected by cancer [18]. In our opinion, this represents the main strength of our study, due to scarcity and limited experience in symptoms evaluation and analysis in patients affected by chronic infectious diseases. Considering PLWH, who represent 19.3% of our series, most of the studies were conducted in the past years, when patients had limited life expectancy due to the scarce availability of highly effective antiretroviral therapy with a high proportion of side effects and low adherence. In relation to their current longer life expectancy, it is important to consider that PLWH is now chronically ill, with the possibility of developing other chronic conditions, such as cardiovascular, oncologic, and neurological diseases, which may bring heavy symptoms to patients with a relevant impact on quality of life. Our HIV-positive patients, in fact, were characterized by a long history of HIV infection and a high rate of intravenous drug use or alcohol abuse; these last aspects can also interfere with symptom perception, evaluation, and response to therapy itself [19]. Moreover, a recent study suggests that many PLWH with chronic pain and depressive symptoms express high levels of pain with deficits in physical function or quality of life despite their use of opioids [20]. These findings highlight the need for a multidisciplinary approach to a good cure also in this setting.

Considering patients with chronic wounds and with bone or prosthetic joint infections, which represent 17% and 44.3%, respectively, of our series, most available data regard specifically diagnosis and etiologic treatment [21-24] rather than symptom burden. In these settings, only by starting with the global assessment of symptoms is it possible to have comprehensive management of these patients who need to be followed for a long time or sometimes lifelong. In fact, in the modern vision of early and simultaneous palliative care [25], this approach should be addressed not only to patients at their end-of-life but especially to those suffering from chronic and progressive diseases, which frequently have a high complexity of concomitant symptoms with a negative impact on their quality of life.

In our study, we demonstrated the utility of ESAS, widely applied and validated in other settings [26-29], as well as in our cohort of patients affected by infectious diseases, even if with some limits. In detail, our study presents at least two points of weakness: first, it is a single-center study, limited to the patients addressed to our department, and second, it has a restricted and heterogeneous sample size, which can lead to some bias. In addition, we chose an approach that allowed us to gather and assess the complexity and intensity of symptoms in patients with advanced chronic or prolonged infectious diseases at a specific point in time but may not fully reflect the dynamic nature of symptom progression in chronic diseases. Even with these limitations, we identified in our patients a high number and high intensity of concomitant symptoms, which were associated with the presence of pain; frequently, these symptoms, for several reasons, are under-reported by patients and under-assessed by physicians, with relevant consequences for patients’ well-being. Our prospective future is to extend our research to a multicenter trial to include a more diversified patient group with a broader range of chronic infectious diseases.

In addition, our results underlined that our population had an HSB and was characterized by a high level of frailty, as represented by the presence of low KPS and a high number of comorbidities and co-medications. These findings are confirmed by another recent study limited to PLWH, where authors found that symptoms were predicted by perceived social support and the number of comorbidities [16]. In addition, another study among adults with advanced, life-limiting illnesses confirmed that taking more medications was associated with higher symptom burden and lower quality of life [30].

## Conclusions

Our preliminary data suggest that it is crucial to identify among chronically infected patients the ones who are at high risk of multiple and complex symptoms in order to make a correct diagnosis and to undertake adequate cures to achieve more comprehensive benefits.
